# Cannibalism and protective behavior of eggs in Arctic charr (*Salvelinus alpinus*)

**DOI:** 10.1002/ece3.8173

**Published:** 2021-09-28

**Authors:** Marilena Frye, Torvald B. Egeland, Jarle Tryti Nordeide, Ivar Folstad

**Affiliations:** ^1^ Faculty of Biosciences and Aquaculture Nord University Bodø Norway; ^2^ Faculty of Education and Arts Nord University Bodø Norway; ^3^ Department of Arctic and Marine Biology UiT – The Arctic University Tromsø Norway

**Keywords:** Arctic charr, cannibalism, egg predation, filial cannibalism, parental care, protective behavior, reproductive behavior

## Abstract

From video recordings of spawning events, we quantified protective and cannibalistic behavior of Arctic charr occurring immediately after spawning. The number of fish cannibalizing on stray eggs was examined regarding (a) whether more than one male shed milt during the spawning event, that is, whether sperm competition occurred, (b) whether the sperm competition included few or many males, that is, the intensity of sperm competition, and (c) the density of fish at the spawning site. Response behavior toward egg cannibalism was also examined among females and dominant males in order to determine any parental investment toward protecting the eggs after spawning. Cannibalistic behavior was seen in almost 50% of the spawnings, and the multiple spawning events showed the highest numbers of fish cannibalizing on eggs. Both the number of males releasing milt and the number of fish approaching the spawning site were positively correlated with egg cannibalism. Sperm competition was, however, not a prerequisite for egg cannibalism. Although we also observed partial filial cannibalism, protective behavior of eggs was seen both among dominant males and females, suggesting that charr actually conduct parental care.

## Graphical abstract

From video recordings of spawning events, we quantified the protective and cannibalistic behavior of Arctic charr occurring immediately after spawning. Cannibalistic behavior was seen in almost 50% of the spawnings. Although partial filial cannibalism was observed, protective behavior of eggs was seen both among dominant males and females, suggesting that charr actually conduct paternal care.

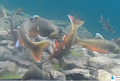

## 1 INTRODUCTION

Historically, the act of cannibalism among fish has been considered an abnormal and maladaptive type of behavior (Persson et al., 2000; Smith & Reay, 1991). Yet, more recent studies have shown that cannibalism is a common phenomenon in many species, and the behavior may have an adaptive value (Manica, 2002; Naumowicz et al., 2017; Smith & Reay, 1991; Svenning & Borgstrøm, 2005). The act of cannibalism involves the killing and consumption of members of the same species regardless of their life stage (Naumowicz et al., 2017; Smith & Reay, 1991), and different forms of cannibalism have been defined over the last decades (Pereira et al., 2017; Smith & Reay, 1991).

The most frequently observed cannibalistic behavior in fish is the consumption of eggs, with the cannibalistic fish taking advantage of the particular vulnerability of this developmental stage (Manica, 2002; Pereira et al., 2017; Smith & Reay, 1991). Although the cannibal is active, the prey in egg cannibalism is clearly passive, since there is no possible escape reaction toward the predator (Smith & Reay, 1991). Egg cannibalism is probably used by individuals to gain energy and other resources (Pereira et al., 2017), resulting in higher growth rates and, in turn, increased fecundity in cannibals compared with noncannibals (Pereira et al., 2017; Van Meyel & Meunier, 2020). Additionally, egg cannibalism may increase survival (Schultner et al., 2013) by, for example, providing resources under periods of food shortage (Pereira et al., 2017; Persson et al., 2000). Additionally, an inclusion of a cannibalistic behavior in an individual's behavioral repertoire might also reduce the number of future competitors for the cannibal's own progeny (Schultner et al., 2013). Besides these obvious benefits, there are surprisingly little data on this type of behavior in external fertilizers.

Sometimes, eggs are even consumed by parents (Smith & Reay, 1991; Tentelier et al., 2011). Such filial cannibalism can be divided into total filial cannibalism, where the whole clutch is consumed, and partial filial cannibalism, where only some of the offspring gets preyed upon (Manica, 2002; Pereira et al., 2017; Smith & Reay, 1991). Filial cannibalism may, because of the unequal investments in zygotes by parents, benefit males more than females (DeWoody et al., 2001). Females will, however, not retain the already‐invested energy in eggs by consuming their own eggs (Kondoh & Okuda, 2002; Manica, 2002, 2004). Males, on the contrary, have larger resource gains and have even been proposed to conduct intrapair parasitism, that is, to trick females into spawning and thus providing them with food resources (Nemtzov & Clark, 1994). Additional male benefits from filial cannibalism may occur when spawning events result in uncertainty about paternity (Pereira et al., 2017). In the best of these cases, cannibalism of eggs with uncertain paternity may reduce the number of future competitors for a male's own offspring (Pereira et al., 2017; Smith & Reay, 1991).

This study adds to the gap of knowledge on egg cannibalism, including filial cannibalism, in external fertilizers using the Arctic charr (*Salvelinus alpinus*) as model. The mating system of Arctic charr can be characterized as lek‐like (Fabricius, 1953; Fabricius & Gustafson, 1954; Fig enschou et al., 2004; Sigurjónsdóttir & Gunnarsson, 1989). That is, males come together at certain spawning sites where they compete over incoming females (Fig enschou et al., 2004; Liljedal & Folstad, 2003; Liljedal et al., 1999; Skarstein & Folstad, 1996). A female that is ready to spawn chooses a suitable part of the lekking site where she stays relatively immobile for several hours. Males have alternative reproductive tactics, and one dominant male always guards the female from other subordinate males by aggressively chasing and biting them (Sørum et al., 2011). The dominant male courts the female frequently by positioning himself alongside her and quivers his body next to hers (Brattli et al., 2018; Fabricius, 1953; Sigurjónsdóttir & Gunnarsson, 1989). Occasionally, the female also quivers along with the male, and this is sometimes followed by “gaping” and simultaneous shedding of gametes (Brattli et al., 2018; Fabricius, 1953; Sørum et al., 2011). Approximately 50 percent of the spawnings, in our study population, occur with one female and one dominant male only, that is, “single spawnings” (Brattli et al., 2018; Sørum et al., 2011), whereas the remaining include sperm competition, that is, “multiple matings” (Sørum et al., 2011).

The Arctic charr has been shown to conduct cannibalism (Smith & Reay, 1991), and cannibalistic charr grow faster than noncannibals (Pereira et al., 2017). Cannibalism in charr has been associated with local environmental conditions, the size ratio of predators versus prey, and the density of alternative conspecific prey (Pereira et al., 2017; Svenning & Borgstrøm, 2005). Thus, the environmental conditions in low‐diversity arctic freshwater habitats with few alternative prey species may have given rise to increased frequencies of cannibalism in these ecosystems (Pereira et al., 2017), and charr is also the most commonly observed cannibal species among the Salmonidae (Pereira et al., 2017).

While most studies on charr are riddled with difficulties in distinguishing between cannibalism and interspecific predation (Pereira et al., 2017; Svenning & Borgstrøm, 2005), this study focuses on the act of spawning and the following egg cannibalism associated with this event, thus clearly differentiating cannibalism from interspecific predation. In our study population, eggs are found in stomach content of dissected fish caught at the spawning grounds during the reproductive period, and both males and females have been observed foraging on eggs after spawning (own observations). We have also observed males, not involved in the actual spawning, eating eggs. Thus, both cannibalism and filial cannibalism occur in the population. Moreover, in multiple spawning events where sperm competition may be intense, paternity of the dominant male may be reduced (Brattli et al., 2018; Egeland et al., 2015), potentially increasing benefits of cannibalism also among dominant males. There will also be more males in and around the spawning site in situations of sperm competitions compared with that of situations of single spawnings (where one male and one female spawn without sneakers), and sperm competition may thus increase the probability of cannibalism.

Here, we examine three questions related to cannibalism in charr: (a) Does the spawning type (i.e., paternal certainty) affect the probability of cannibalism?; (b) Does, additionally, the number of fish (i.e., potential cannibals) approaching the spawning site affect the number of fish showing cannibalistic behavior; And (c) does the spawning female or the spawning dominant male show any behavior that could be related to egg protection?

## 2 METHODS

We reanalyzed underwater film recordings from spawning grounds number 2 and 3 in Fjellfrøsvatn, Northern Norway (see Fig enschou et al., 2004), where spawning situations already had been identified on the videos in an earlier study (Brattli et al., 2018). There are two populations of charr and one population of trout (*Salmo trutta*) in Lake Fjellfrøsvatn (Klemetsen et al., 1997), yet the spawning fish at the locations used in this study are all from the same, locally reproducing, charr population (Fig enschou et al., 2004). The analyzed video material was collected between 17 and 25 September 2016 (Brattli et al., 2018) and included a total number of 109 spawning events that were analyzed in chronological order using QuickTime player. All recordings were from eight wide angle GoPro Hero 3 and 4 (types silver, plus, and black) cameras equipped with waterproof housing. The video quality was set to 1,080p and 60 frames per second (for more details, see Brattli et al., 2018). The cameras were deployed pointing toward females that appeared to be preparing to spawn. The recording lasted as long as the battery allowed (from 90 to 270 min). Both sounds and videos were recorded (Brattli et al., 2018), but for our purposes, only the videos were used to analyze the cannibalistic and protective behavioral pattern in the Arctic charr.

### 2.1 The analysis

The first step of the analysis was defining the different behavioral patterns of interest. Based on these definitions, each spawning event was analyzed separately in chronological order, thereby enabling counts of the number of fish showing the different behavioral patterns. The procedure also enabled identification of whether the females and dominant males showed filial cannibalism. As the number of fish approaching the spawning site could influence the number of fish also cannibalizing eggs, the number of fish approaching the spawning site in every spawning event was separately counted (see Brattli et al., 2018, for more details). Other important data, such as the number of males releasing milt and the spawning type, were obtained from our previous datasets collected from the same videos (Brattli et al., 2018). The video material was repeatedly evaluated to gather the most accurate information.

### 2.2 Definitions of different behaviors

Brattli et al. (2018) used four definitions describing different types of spawning behavior.

1. The stationary, close to the bottom substrate laying, female shows signs of an erected anal fin and is pointing the upper body slightly upward.
2. Both males, guarding and sneaker males, approach the female from behind to court her. The quivering is initiated by the male's head touching the female's tail. Females also respond with a quivering slightly after the male's body touches hers.
3. The intensity of the quivering increases until gaping. Both the female and the male show this type of behavior, but the female often gapes first. The gametes are released when the mouth is fully opened. Both sexes swim slightly up and forward at the same time as their mouths open and their heads are lifted.
4. After releasing the milt, the female and male separate and both are returning to the spawning ground to defend it from other fish.

Sørum et al. (2011) also suggest that females return to the spawning ground immediately after spawning to cover and protect the spawned eggs. In the present study, all four behaviors, in right sequence, were used as criteria for the composite behavior termed “reproductive event.”

In addition to the previously described mating behavior, the following definitions were added:

#### 2.2.1 Cannibalistic behavior

Fish are reaching for and prey upon eggs. They sometimes take an almost vertical position in the water column toward the sediment or eggs laying at the bottom. Stray eggs floating in the water are consumed without any of the previously mentioned behavioral patterns.

#### 2.2.2 Female protective behavior

The female hovers above the spawning ground and eventually chases away intruders, presumably to protect the eggs. The female is specifically approaching the fish that are trying to prey on the eggs by ramming into their sides and trying to bite them.

#### 2.2.3 Dominant male protective behavior

The dominant male chases away competitors, bites their fins, and stays close to the female to guard her. The male often tries to swim through the group of fish cannibalizing on the eggs and at the same time tries to stay close to the spawning ground, where the eggs are lying.

#### 2.2.4 Fish approaching the spawning ground

Approaching fish are turning toward the spawning couple and start approaching and taking up speed toward the spawning ground. In this study, the distance of the approaching fish was disregarded and only fish turning, approaching, and picking up speed within 3 s after the actual spawning started, that is, 3 s after the quivering started, were included.

Every criterion in each of the points above had to be fulfilled in a particular video sequence in order to include this sequence in the analyses.

### 2.3 Statistics

**FIGURE A1:**
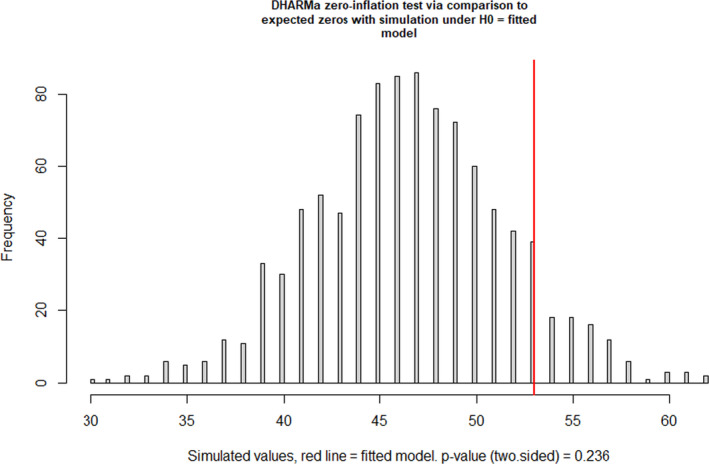
Test for zero inflation from the DHARMa package

**FIGURE A2:**
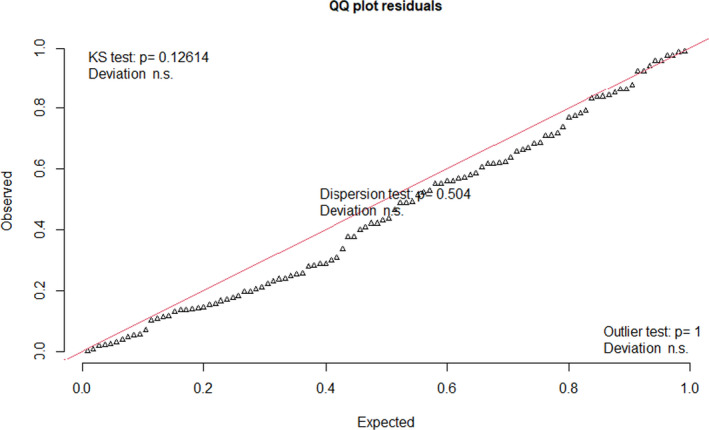
Q–Q plot including distribution test (KS test), dispersion test, and outlier test

The computing program R v. 4.0.3 (R Core Team, 2020) was used to perform all necessary statistical analyses. A generalized linear mixed model (GLMM) from the package glmmTMB v. 1.0.2.1 (Brooks et al., 2017) was used to calculate the effect of spawning type (multiple or single), number of approaching fish, and number of males releasing milt on the number of egg‐cannibalizing fish. Model selection was based on an information‐theoretic approach (AIC: Burnham & Anderson, 2001). That is, we fitted GLMMs to the data with Poisson, Conway–Maxwell–Poisson, and negative binomial distributions on the conditional models and the null models (Brooks et al., 2017). Spawning type, number of approaching fish, and number of males releasing milt were entered as fixed factor and Female ID as random factor. Based on the AICtable (Table A1), the most parsimonious model had a Conway–Maxwell–Poisson distribution with spawning type and number of approaching fish as fixed factors. The model was screened for collinearity between predictor variables by evaluating the variance inflation factor (VIF) using the performance R package version 0.7.3 (Lüdecke et al., 2021). VIF scores were <2 indicating low collinearity. Model validation was carried out using the DHARMa package (Figures A1 and A2; Hartig, 2021). The ggplot2 R package version 2.1.0. was used to visualize the raw data (Wickham, 2009).

## 3 RESULTS

### 3.1 Egg cannibalism and spawning type

**FIGURE 1:**
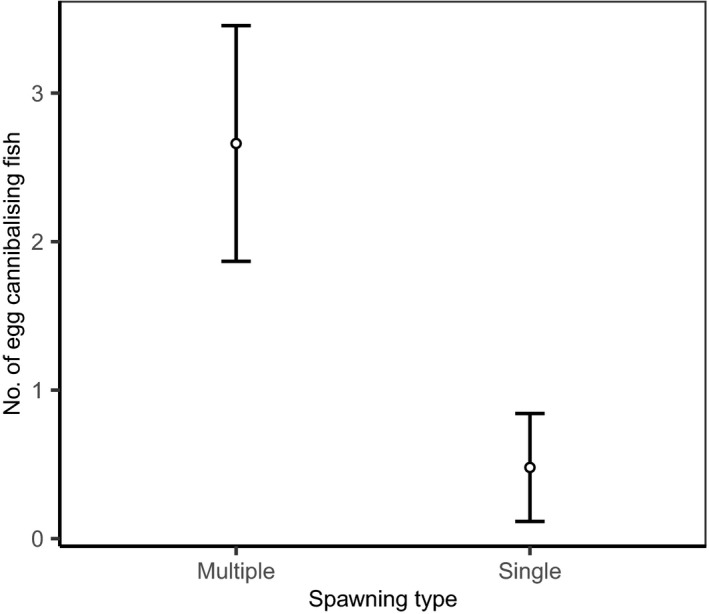
Number of egg‐cannibalizing fish (mean ± 95% CI) in multiple spawning events (*N* = 58) and single spawning events (*N* = 51)

Egg cannibalism was observed in 48 (46.15%) of the 104 analyzed reproductive events. A total number of 48 single spawning events and 56 multiple spawning (i.e., with sperm competition) events were included, and egg cannibalism was present in 66.1% of the multiple spawning events, whereas 29.1% of the single spawning events had egg cannibalism. The frequency of egg cannibalism was significantly higher in multiple spawning events than in single spawning events (binomial test comparing two proportions, 95% CI = 0.17–0.57, *χ* ^2^ = 12.6, *p* < .001). The difference in the number of cannibalizing fish between the two spawning types, single and multiple spawning, was statistically significant (GLMM, *χ* ^2^ = 6.76, *p* < .001; see Table A2 for parameter estimates). Additionally, there were 82% less egg‐cannibalizing fish found in single spawning events compared with that of multiple spawning events (Figure 1).

### 3.2 Egg cannibalism and number of approaching fish

**FIGURE 2:**
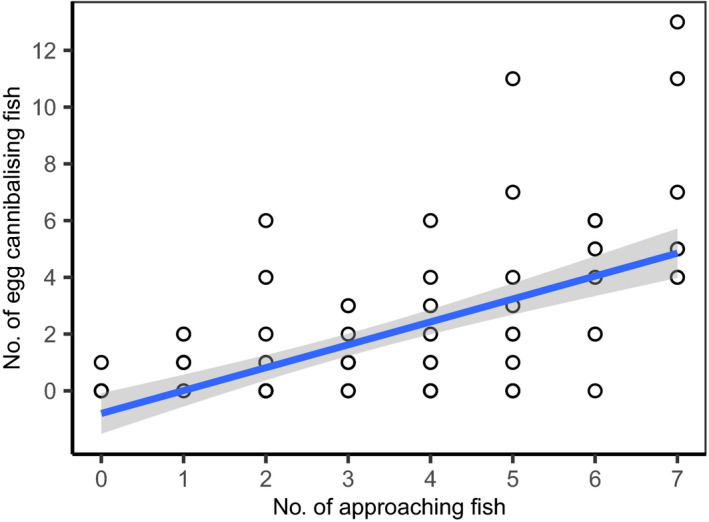
Number of fish approaching the spawning site plotted against the number of egg‐cannibalizing fish in a spawning event. These approaching fish did not engage in sperm competition. Blue line represents the regression line including 95% confidence interval (gray ribbon)

The number of approaching fish and the number of cannibalistic fish were also positively correlated with an estimate of GLMM (*χ* ^2^ = 32.46, *p* < .0001; see Table A2 for parameter estimates). So, the more the fish approaching the spawning ground, the more the fish preyed on the eggs (Figure 2). An increase in the number of approaching individuals may thus result in an increase in the number of cannibalistic fish with a factor of 1.51. That is, if this is a causal relationship, one more individual approaching the spawning site will on average result in 1.51 more individuals cannibalizing on eggs.

### 3.3 Filial cannibalism

Females preyed on their own stray eggs in eight out of the 104 spawning events. Of these eight events, six were multiple spawning events where an average of 5.3 other individuals also ate eggs (range 0–12). The dominant males only preyed on eggs in two out of 104 cases in which both were multiple spawning events.

### 3.4 Protective behavior

65% of the females showed protective behavior of eggs, whereas 75% of the dominant males showed protective behavior of eggs immediately after spawning. The spawning sneakers were not considered in these evaluations.

**FIGURE 3:**
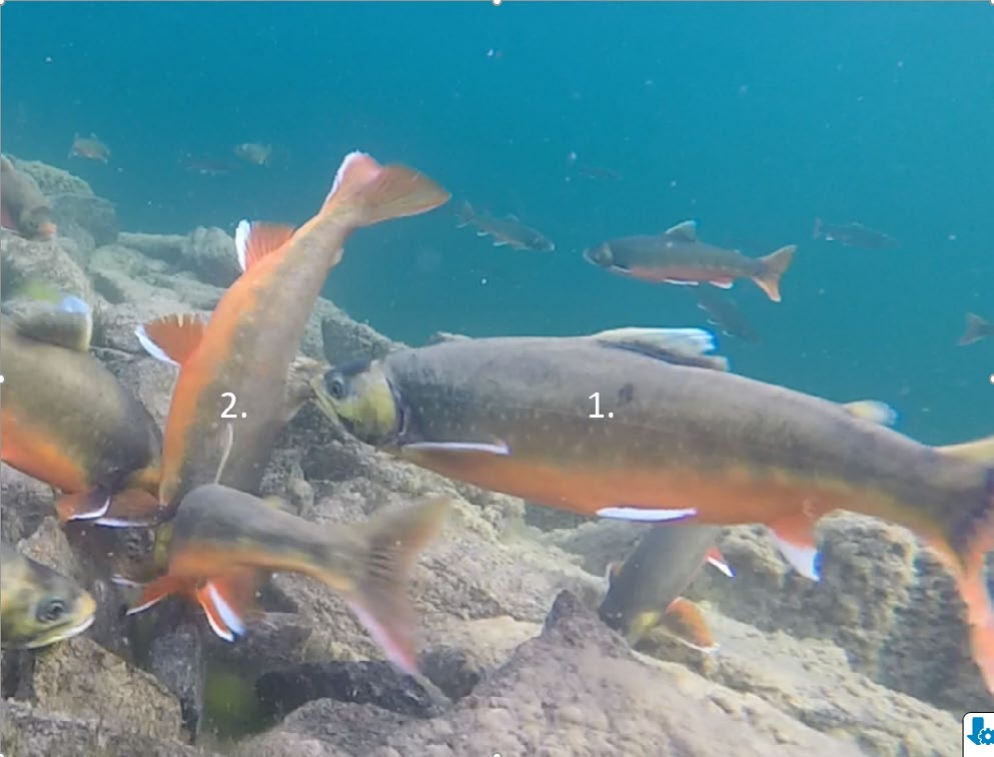
Immediately after spawning, the dominant male (1.) bite another individual's (2.) dorsal fin, possibly to protect against egg cannibalism. Note that the substrate for oviposition is stones, not gravel (the female is not present in the picture)

**FIGURE 4:**
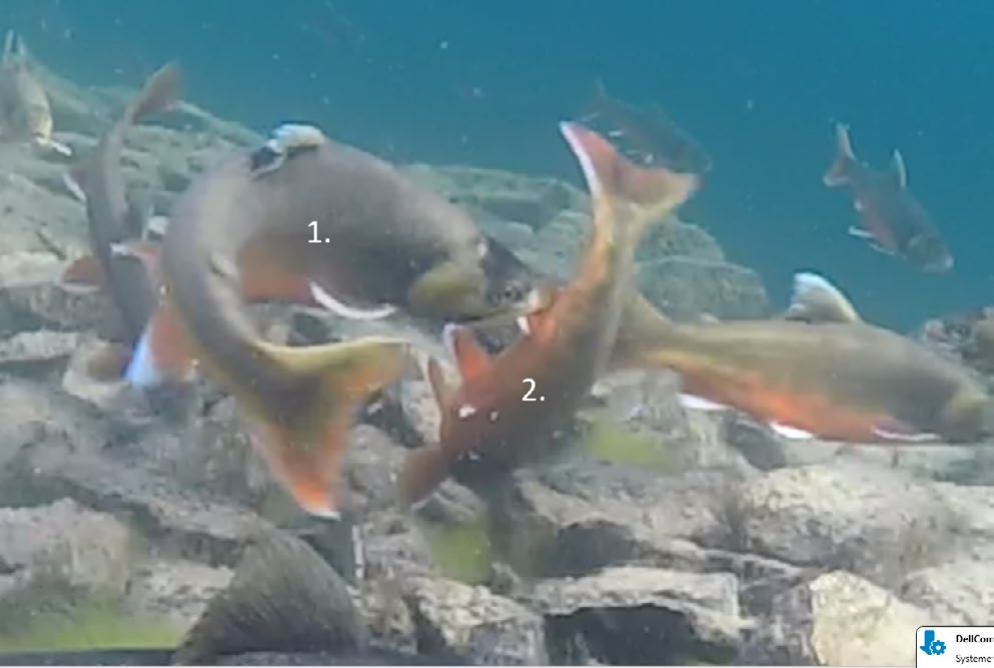
Dominant male (front left) bites another individual's (front middle) anal fin immediately after spawning. The behavior may be targeted to protect the spawned eggs against predation and would, in case, suggest paternal care in charr

The following Figure 3 and Figure 4 show that the male's protective behavior can be aggressive, including biting other male's dorsal and anal fins.

## 4 DISCUSSION

In general, the results from our study population reveal some interesting behaviors among Arctic charr: Egg cannibalism occurs in almost half of the recorded spawning events, it intensifies, as expected, with increased sperm competition and with increased numbers of approaching peripheral fish. Additionally, although both males and females show filial cannibalism, both the dominant males and the females have behaviors that could be interpreted as protective against egg cannibalism.

Egg cannibalism was observed in 46% of the recorded spawnings. Comparable numbers have been reported for *S. trutta* (Aymes et al., 2010), where egg cannibalism of broods buried in gravel was observed in 25.3% of events, whereas egg picking of stray eggs occurred in 66.3% of the spawnings. Moreover, the frequency of cannibalistic behavior differs between species (Pereira et al., 2017), and between populations within species (Aymes et al., 2010). Two variables quantified here seem important for such variation—spawning types and the overall number of approaching individuals.

### 4.1 Different spawning types

Cannibalism of stray eggs was not conducted irrespectively of whether spawnings were single or multiple; the multiple events had higher number of cannibals than single events. A simple explanation for this would be an observational bias, that is, that when more fish are involved in the spawning, the higher density of fish increases the spectacularity of the individual competition postspawning and eases our observation of cannibalism taking place. Yet, this is an unlikely explanation as the high resolution of our video recordings will reveal cannibalism irrespective of the spectacularity of the postspawning competition. Yet, reduced paternal certainty may increase fitness benefits from egg consumption for all males involved in the act of spawning, and it may in the best of cases also reduce intraspecific competition in the next generation.

Yet, is reduced paternity certainty the cause of egg cannibalism? Probably not. The increased density of males at the spawning site under multiple spawnings will lead to a higher encounter rate between stray eggs and individual fish. This density dependence will alone produce higher frequencies of cannibalism under multiple spawnings (Smith & Reay, 1991). Although sperm competition clearly increases the frequency of cannibalism, its presence is not a precondition for egg cannibalism. That is, although fewer cannibals are associated with single spawning events, also these events show cannibalism. Additionally, increased egg cannibalism is also associated with increasing amounts of approaching peripheral fish unable to arrive in time for the actual sperm competition. These approaching fish, that did not release milt, do not benefit from increased reproductive success, only nutritionally from consumption of stray eggs.

### 4.2 Filial cannibalism

Filial cannibalistic behavior in female Arctic charr has to our knowledge not been documented before. Our observations cannot, however, distinguish whether the female consumed the egg(s) or whether they only engulfed them to protect them from other cannibals. The former is, however, most likely as we never observed attempts to relocate eggs by the females. The eggs seen engulfed by the females are discovered floating in the water column before being preyed upon and they are easy prey for all surrounding fish. Thus, the filial cannibalism, most often seen in multiple spawnings with considerable numbers of surrounding fish, may instigate females to make the best out of an undesirable situation and consume eggs that would be eaten anyway. Because unprotected eggs are easily preyed upon, they may be considered to have low reproductive value, and such eggs have also previously been observed eaten by females (Vallon & Heubel, 2016). The few observations of filial cannibalism among males also occurred under sperm competition (i.e., under paternity uncertainty), and it should be noted that male differentiation between own and foreign progeny in these cases would be highly unlikely to occur just two seconds after spawning.

### 4.3 Protective behavior

Seventy‐five percent of dominant males and 65% of females show protective behavior of eggs after the spawning event. The behavioral repertoire used by the dominant males seems similar to their prespawning behavior. Comparable protective behavior has been reported in the brown trout (*S. trutta*), and in this species, the defensive behavior is also negatively related to the probability of egg cannibalism (Tentelier et al., 2011). Female charr also approached and chased away fish trying to cannibalize on their eggs by chasing and biting their subdominant male conspecifics at their fins or ramming them into their lateral areas (Figures 3 and 4).

The high frequency of protective behavior among males might be surprising, since males may be more uncertain about paternity. On the contrary, the male has invested considerable resources in courtship and protection of the fertilization itself at the time of spawning. Additionally, because of benefits from sex‐specific reproductive behaviors, where dominant males constantly attack subordinates, aggressive behavior may in general be more easily triggered in males than in females throughout the entire spawning cycles. In either case, the protective behavior against cannibalism from conspecific subdominant males from both females and the dominant males extends the postcopulatory competition beyond sperm competition. The behavior seems to represent an active support of the reproductive investments from both males and females. Thus, contrary to all previous descriptions, charr in our particular population seem to conduct parental care.

## CONFLICT OF INTEREST

None declared.

## AUTHOR CONTRIBUTIONS

**Marilena Frye:** Conceptualization (lead); Data curation (equal); Formal analysis (equal); Investigation (lead); Methodology (equal); Project administration (lead); Validation (equal); Visualization (equal); Writing‐original draft (equal); Writing‐review & editing (equal). **Torvald B. Egeland:** Conceptualization (equal); Data curation (lead); Formal analysis (lead); Investigation (equal); Methodology (equal); Project administration (equal); Resources (equal); Validation (equal); Visualization (equal); Writing‐original draft (equal); Writing‐review & editing (equal). **Jarle Tryti Nordeide:** Conceptualization (equal); Formal analysis (equal); Investigation (equal); Methodology (equal); Project administration (equal); Validation (equal); Visualization (equal); Writing‐original draft (equal); Writing‐review & editing (equal). **Ivar Folstad:** Conceptualization (equal); Data curation (equal); Formal analysis (equal); Investigation (equal); Methodology (equal); Project administration (equal); Validation (equal); Visualization (equal); Writing‐original draft (equal); Writing‐review & editing (equal).

### OPEN RESEARCH BADGES

This article has earned an Open Data Badge for making publicly available the digitally‐shareable data necessary to reproduce the reported results. The data is available at https://doi.org/10.18710/3QNDQ7.

## Data Availability

All data presented in this study (including behavioral data and R script) are available at DataversNO https://doi.org/10.18710/3QNDQ7. The video recordings that the behavioral data are based upon are available in “The spawning behaviour of Arctic charr video collection” on DataverseNO https://doi.org/10.18710/HTM6‐F146.

## APPENDIX A

### MODEL SELECTION BASED ON AKAIKE’S INFORMATION CRITERION

**TABLE A1:** AICtable showing the most parsimonious (dAIC > 5) model to predict the effect of spawning type on the number of cannibalistic fish

Response	Predictor(s)	Distribution	*df*	dAIC
Number of cannibalistic fish	ST, NOAF	Conway–Maxwell–Poisson	5	0.0
	ST, NOAF, NOMRM	Conway–Maxwell–Poisson	6	2
	ST, NOAF	Negative binomial	5	2.1
	ST, NOAF, NOMRM	Negative binomial	6	4.1
	NOAF, NOMRM	Conway–Maxwell–Poisson	5	4.4
	NOAF	Conway–Maxwell–Poisson	4	4.9

Abbreviations: NOAF, number of approaching fish, NOMRM, number of males releasing milt; ST, spawningtype.

#### Model summary

**TABLE A2:** Parameter estimates from a Conway–Maxwell–Poisson generalized linear mixed‐effects model (log link function) testing the effect of spawning type and number of approaching fish on number of cannibalistic fish

Response	Predictor	Estimate	*SE*	95% CI	*p*
Number of cannibalistic fish	Intercept	−0.89	0.34	−1.46 to −0.10	.025
	Spawning type—single	−0.83	0.31	−1.45 to −0.20	.009
	No. of approaching fish	0.38	0.01	0.24 to 0.51	<.0001
